# Supplementary material for: Representing high throughput expression profiles via perturbation barcodes reveals compound targets
Source: PLoS Comput Biol. 2017 Feb 9;13(2):e1005335. doi: 10.1371/journal.pcbi.1005335 (PMC5300121; doi:10.1371/journal.pcbi.1005335)
Supplement: S1 Text — Figures showing sample correlation, sample space depiction, and details of prospective prediction results. (PDF) [file pcbi.1005335.s001.pdf]

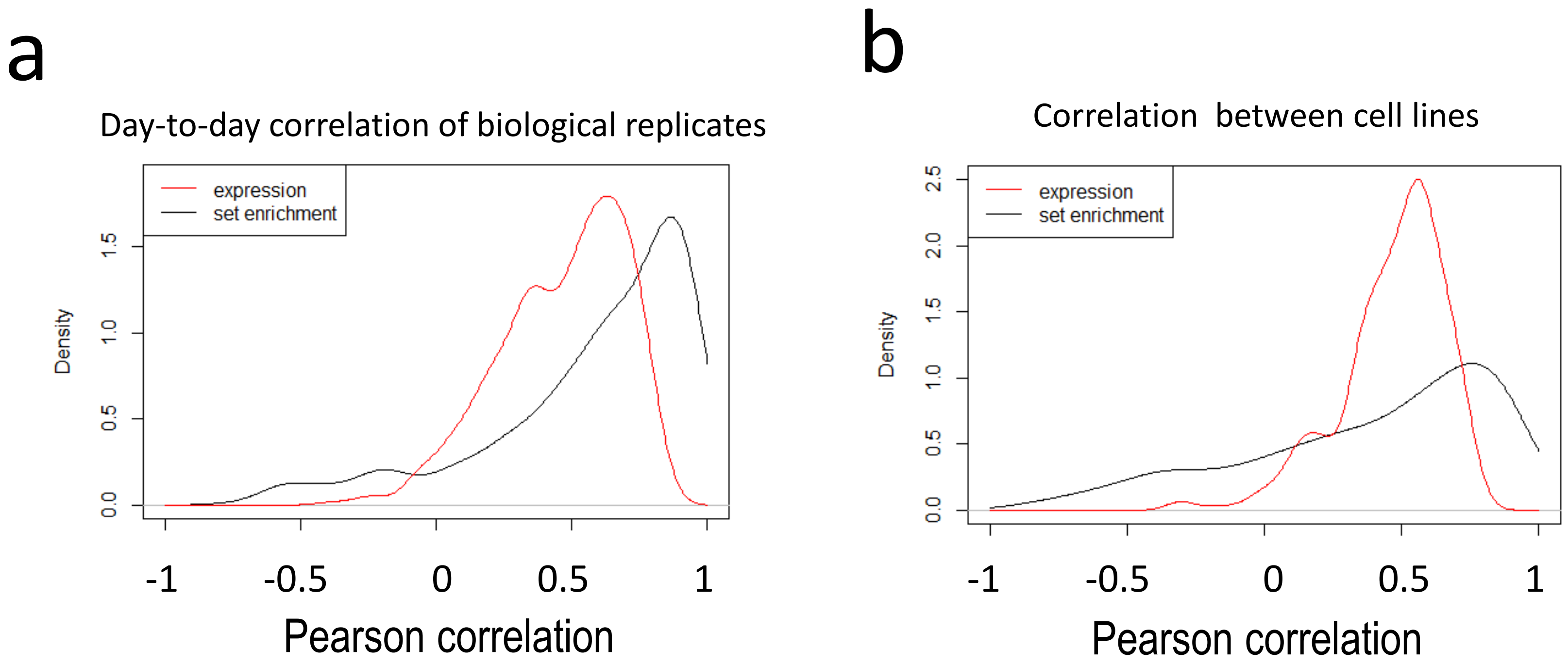

Fig A. Correlation of profiles of samples treated with the same compound and dose on different days **(a)** or in different cell types **(b)**. The red line indicates z-scores of gene expression changes, and the black line indicates gene set enrichment scores derived from the z-scores and a library of experimentally derived gene sets. The distribution is smoothed using a Gaussian kernel density estimate.

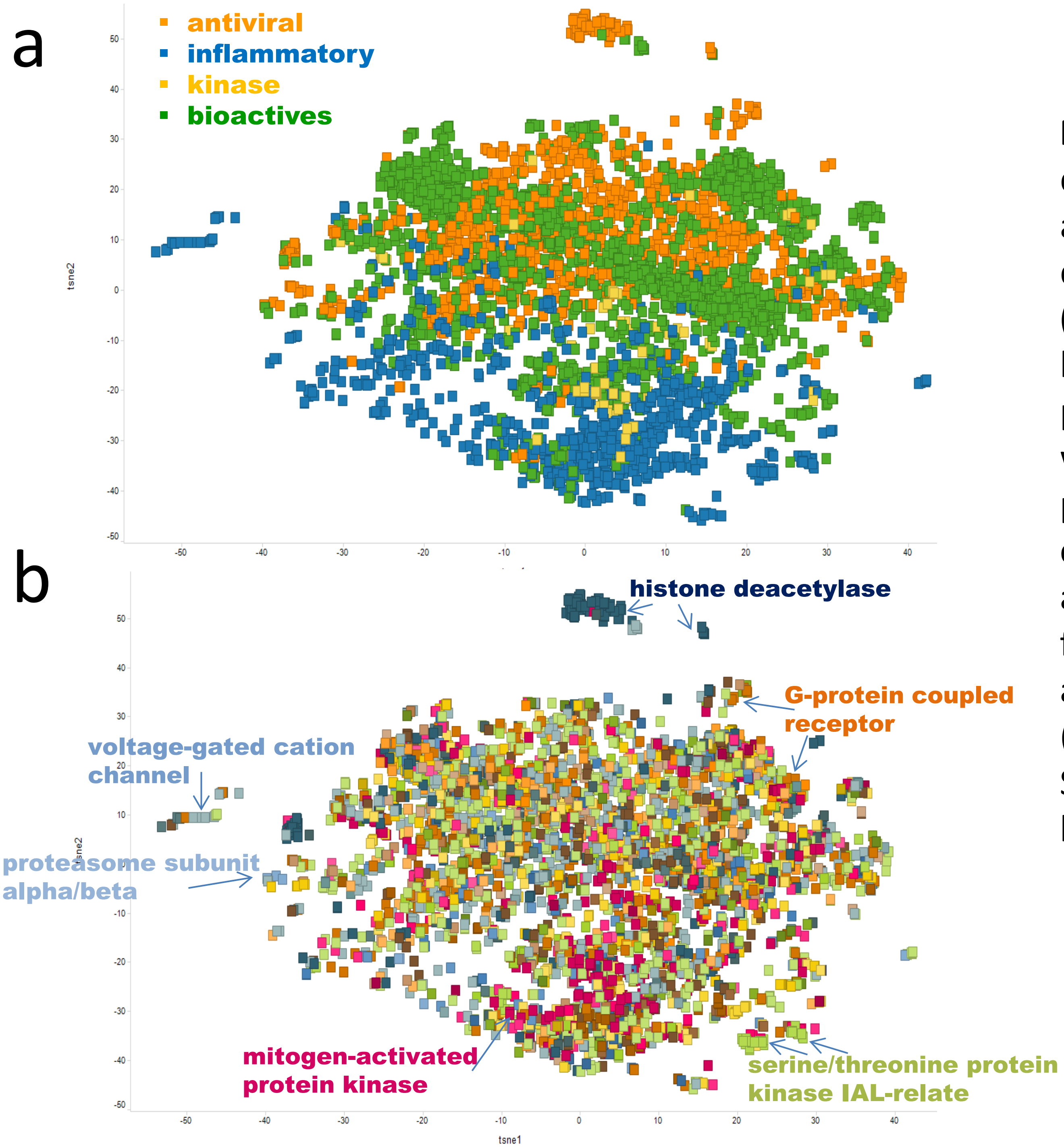

Fig B. Overall shape of dataset used in this study, as summarized by t-SNE dimensionality reduction. **(a)** Sample points colored by provenance, i.e. the project from which they were derived. **(b)** Sample points colored by compound's highest affinity target's PANTHER family (Thomas, Paul D., et al. Genome research 13.9 (2003): 2129-2141). Selected families highlighted.

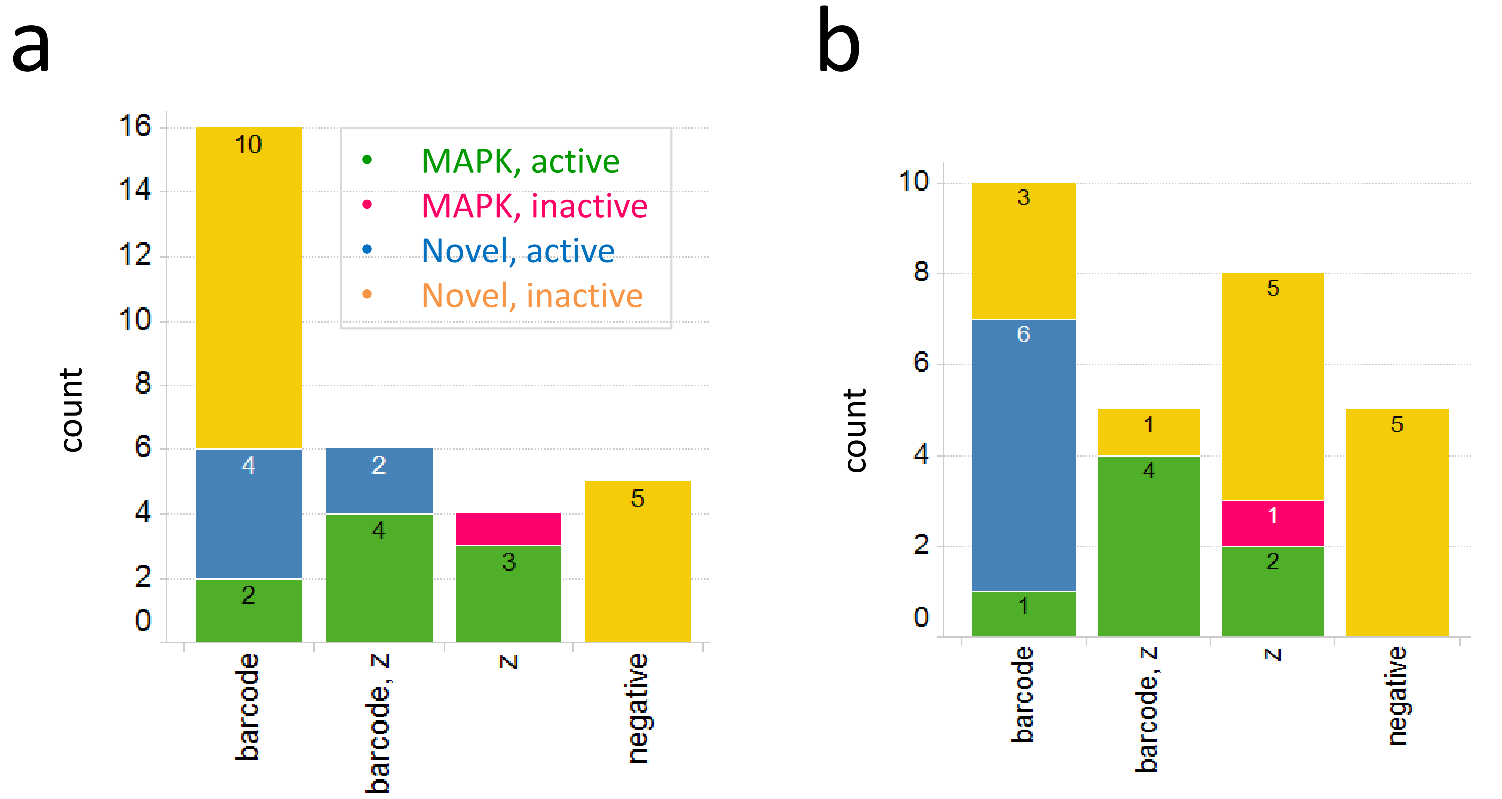

Fig C. Activity prediction results. **(a)** Candidates selected based on t-SNE maps of z-scores, barcodes, or both, are shown along with activity in the EGF/AP1 reporter assay. Green denotes known MAPK actives that were confirmed active in the reporter assay, red was known active that did not show activity. Blue denotes novel compounds that showed MAPK activity, yellow indicates novel compounds with no significant activity. Samples labeled 'negative' were negative control compounds selected to be dissimilar to MAPK profile, all were confirmed inactive in the reporter assay. **(b)** Candidates selected based on the native datasets (978-dimensional) z-scores, (100-dimensional) barcodes, or both, are shown along with the observed activity in the EGF/AP1 reporter assay.
